# Redox Control of Signalling Responses to Contractile Activity and Ageing in Skeletal Muscle

**DOI:** 10.3390/cells11101698

**Published:** 2022-05-20

**Authors:** Malcolm J. Jackson, Natalie Pollock, Caroline Staunton, Samantha Jones, Anne McArdle

**Affiliations:** MRC-Versus Arthritis Centre for Integrated Research into Musculoskeletal Ageing (CIMA), Department of Musculoskeletal and Ageing Science, Institute of Life Course and Medical Sciences, University of Liverpool, Liverpool L7 8TX, UK; natzpol@liverpool.ac.uk (N.P.); staunton@liverpool.ac.uk (C.S.); samantha.jones2@liverpool.ac.uk (S.J.); mdcr02@liverpool.ac.uk (A.M.)

**Keywords:** muscle, contraction, sarcopenia

## Abstract

Research over almost 40 years has established that reactive oxygen species are generated at different sites in skeletal muscle and that the generation of these species is increased by various forms of exercise. Initially, this was thought to be potentially deleterious to skeletal muscle and other tissues, but more recent data have identified key roles of these species in muscle adaptations to exercise. The aim of this review is to summarise our current understanding of these redox signalling roles of reactive oxygen species in mediating responses of muscle to contractile activity, with a particular focus on the effects of ageing on these processes. In addition, we provide evidence that disruption of the redox status of muscle mitochondria resulting from age-associated denervation of muscle fibres may be an important factor leading to an attenuation of some muscle responses to contractile activity, and we speculate on potential mechanisms involved.

## 1. Introduction

Loss of skeletal muscle occurs in many pathophysiological situations (e.g., disuse, ageing, weightlessness) and also secondary to multiple disorders, such as cancer and chronic obstructive pulmonary disease (COPD). Muscle loss contributes to reduced mobility, lack of independence, and poor recovery. Skeletal muscle rapidly adapts to changes in everyday use by increasing or decreasing muscle mass and/or length. Extreme examples of disuse-induced muscle loss include exposure to microgravity, where a substantial decrease in muscle mass occurs in all skeletal muscles within 17 days [1], and extended bed rest, which is commonly used as an experimental model of muscle disuse in mechanistic experiments [2]. Loss of protein homeostasis (proteostasis) has been extensively reported as the final common event leading to loss of muscle mass [3,4]. Multiple mechanisms controlling muscle proteostasis are recognised [4], but understanding of the initiating signals controlling the activation of these proteostatic pathways remains incomplete.

Regular exercise or training is essential for the maintenance of skeletal muscle mass and function at all stages of the life course. Substantial research has identified mechanisms by which exercise, or contractile activity, acts to maintain skeletal muscle mass and function, and many key molecular and biochemical pathways have been identified, but knowledge is incomplete on specific changes that occur in muscles during exercise to initiate the signalling pathways leading to these adaptations. Reactive oxygen species (ROS) have been proposed as key factors that initiate adaptive changes in contracting skeletal muscle, with hydrogen peroxide (H_2_O_2_) thought to play the major role [5,6,7]. It is important to recognise that these roles of H_2_O_2_ are as a signalling molecule and reflect H_2_O_2_ stimulation of redox-signalling pathways. This is a role of H_2_O_2_ in physiological processes which is distinct from “oxidative damage”. The recognition of both physiological and pathological roles of H_2_O_2_ and other ROS has led to a redefinition of “oxidative stress”, with physiological processes being described as “oxidative eustress” and pathological or damaging events described as “oxidative distress” [8]. There are multiple existing reviews of the potential roles of oxidative distress or oxidative damage in skeletal muscle, and interested readers are referred to References [9,10,11,12].

## 2. Muscle Weakness during Ageing Is Due to Loss and Weakness of Muscle Fibres and Linked to Loss of Motor Units

In human beings, ageing leads to a reduction in skeletal muscle cross-sectional area of 25–30% and strength by 30–40% [13]. This leads to an inability to undertake everyday tasks, increasing risk of falls and loss of independence [14]. Both loss of muscle fibres and atrophy of fibres contribute to the reduction in muscle mass and function in ageing humans and rodents [15,16,17]. The age-related changes in rodents follow a similar pattern to those in humans, supporting their use as relevant models [18,19]. Motor unit loss is also seen with ageing in both humans and rodents [20,21], and the number of motor neurons is reported to decrease in both humans and rodents with ageing, by 25–50% [22,23]. Studies from our laboratory reported that full denervation of ∼15% of muscle fibres was seen in muscles of old mice, with disruption of the NMJ apparent in ∼80% of the muscle fibres [24].

## 3. Importance of Exercise in Maintaining Muscle Mass

Different forms of exercise induce positive adaptations in skeletal muscle, including an increase in aerobic capacity, increased muscle force generation, increased mass, and decreased fatigability [25]. The importance of these processes becomes increasingly important in older people [26,27]. H_2_O_2_ has been proposed as a key primary factor that initiates adaptive changes in contracting skeletal muscle through redox-signalling pathways [5,6,7].

## 4. ROS Are Generated by Various Potential Sources in Skeletal Muscle during Contractile Activity

**Figure 1:**
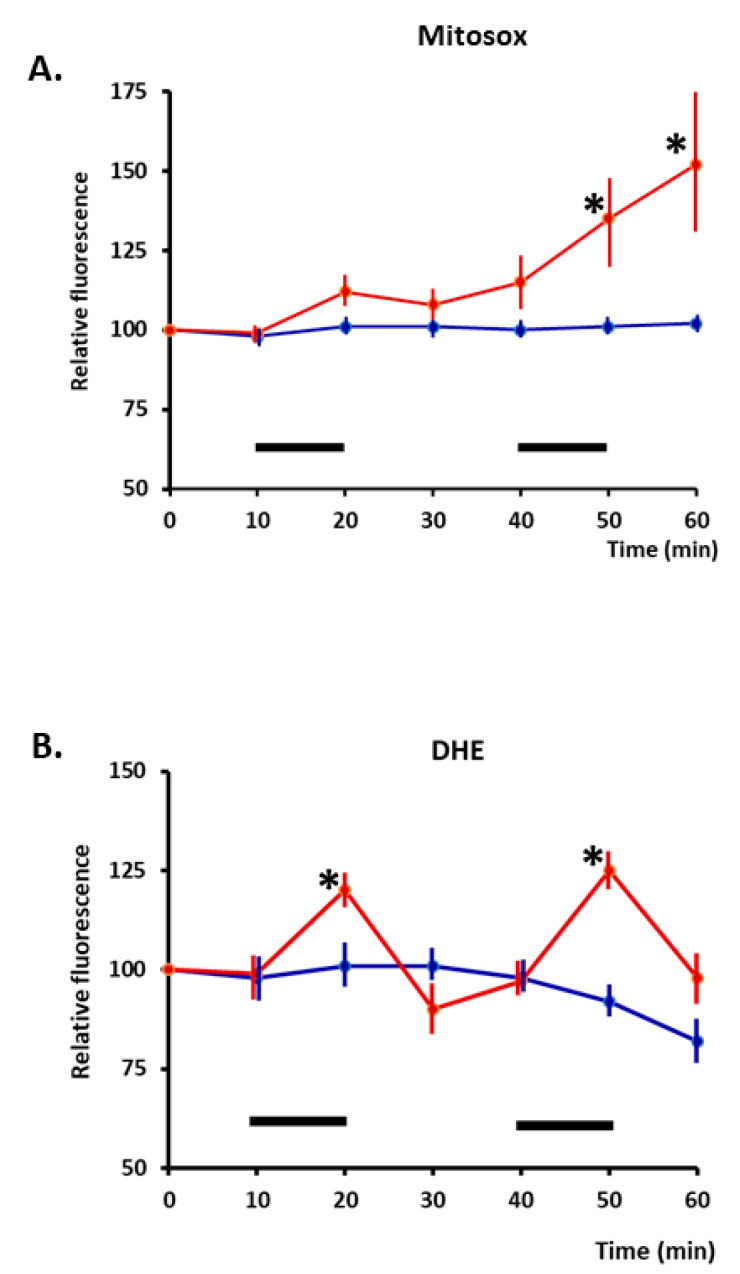
(**A**). Time course of changes in relative fluorescence from skeletal muscle fibres loaded with MitoSox red (localised to mitochondria). Fibres were either maintained at rest (blue line) or subjected to two periods of electrically stimulated contractions (red line) during the time periods denoted by a black bar. (**B**). Fibres loaded with DHE (localizes to cytosol): time course of changes in relative fluorescence is shown. Fibres were either maintained at rest or subjected to two periods of electrically stimulated contractions, with the stimulation periods denoted by black bars. * *p* < 0.05 compared with non-stimulated fibres at the same time point (*n* = 6–7 for all groups). Redrawn from [32], where full experimental details can be found.

Contractile activity increases superoxide and nitric oxide (NO) in skeletal muscle fibres, and these species lead to the formation of secondary ROS and reactive nitrogen species [5,28,29]. Initial studies of the sources of superoxide generation identified the mitochondrial electron transport chain as a potential source [30], but studies of short-duration (15 min) contractions that induced adaptive responses indicated that NADPH oxidase was the likely source of superoxide generation leading to the formation of H_2_O_2_ [31]. Few detailed studies of longer-duration contractions have been undertaken, but Pearson et al. [32] compared the response of dihydroethidium (localised to the cytosol) and mitoSox (dihydroethidium localised to mitochondria) in isolated fibres from the mouse flexor digitorum brevis (FDB) and concluded that cytosolic superoxide increased rapidly during 10 min of isometric contractile activity, but after two periods of contractions, mitochondrial superoxide was also increased (Figure 1). H_2_O_2_ has been increasingly recognised as a physiological signalling molecule [33,34,35], and signalling by H_2_O_2_ appears to occur via redox modifications of specific residues in proteins, particularly redox-sensitive cysteines [36,37].

## 5. ROS Stimulation of Adaptations to Exercise Appears to Occur by Redox-Regulated Signalling Pathways

Data from studies that have attempted to reduce the actions of ROS in exercising muscle by interventions using very high levels of single antioxidant nutrients, or mixtures of antioxidants, have been contradictory [38,39], but in some experiments, the antioxidants were found to inhibit cytoprotective responses (e.g., exercise-induced increase in heat shock and other stress proteins) [40,41], reduce mitochondrial biogenesis [42,43,44], prevent an increase in muscle insulin sensitivity [42], and inhibit the release of cytokines and inflammatory mediators [45].

In parallel to these studies, several key signalling pathways involved in skeletal muscle responses to contractile activity have been recognised as redox-regulated. Example pathways include mitogen-activated protein kinases (MAPK), protein tyrosine phosphatases (PTP), peroxisome proliferator-activated receptor gamma (PPAR-γ), and nuclear factor-κB (NF-κB) [46,47,48,49,50], all of which are increased in muscles by exercise and inhibited in some models by exogenous antioxidants.

Studies in vitro have demonstrated that H_2_O_2_ can activate specific signalling pathways which are also activated in muscle by contractile activity in vivo, and this has been considered by some authors as evidence for redox regulation playing a role in activation of these pathways during exercise in vivo. However, we have argued that the in vivo concentrations of H_2_O_2_ are not sufficiently high to directly activate key signalling molecules. [46]. Thus, key cysteines in the signalling molecules examined are relatively unreactive with H_2_O_2_, and concentrations of H_2_O_2_ that have been shown to activate them are in the range of 100−1000 µM. In comparison, we have calculated that muscle intracellular H_2_O_2_ concentrations are in the order of 1–10 nM [51], and during contractions may increase to a maximum of 100 nM [46,52].

## 6. Are Additional Effector Proteins Required to Allow H_2_O_2_ to Stimulate Signalling Pathways at the Intracellular Concentrations Calculated to Occur in Contracting Muscle Fibres In Vivo?

It has been argued that oxidation of key signalling molecules may be facilitated by the proximity of the target protein to the source of generation of H_2_O_2_, where local concentrations may be increased [53,54], but computational modelling does not support this [55]. Alternatively, the “floodgate” hypothesis proposes that local scavengers of H_2_O_2_ (such as peroxiredoxins) become rapidly oxidised and inactivated, subsequently allowing a local increase in the local H_2_O_2_ concentration to micromolar concentrations [56] to permit oxidation of relatively unreactive target signalling molecules.

An alternate possibility requires the use of highly oxidisable effectors of redox signalling that allow transmission of the oxidising equivalents from H_2_O_2_ to less oxidisable target signalling molecules. Only a small number of proteins appear capable of undertaking a reaction with H_2_O_2_ at the concentrations found in the muscle cytosol, including peroxiredoxins (Prx), thioredoxins (Trx), glutathione peroxidases, and catalase. In muscle fibres, mass spectrometry data indicate that Prx concentrations are much higher than the other proteins [57].

## 7. Peroxiredoxins as Effectors of H_2_O_2_ Signalling in Skeletal Muscle Contractions

**Figure 2:**
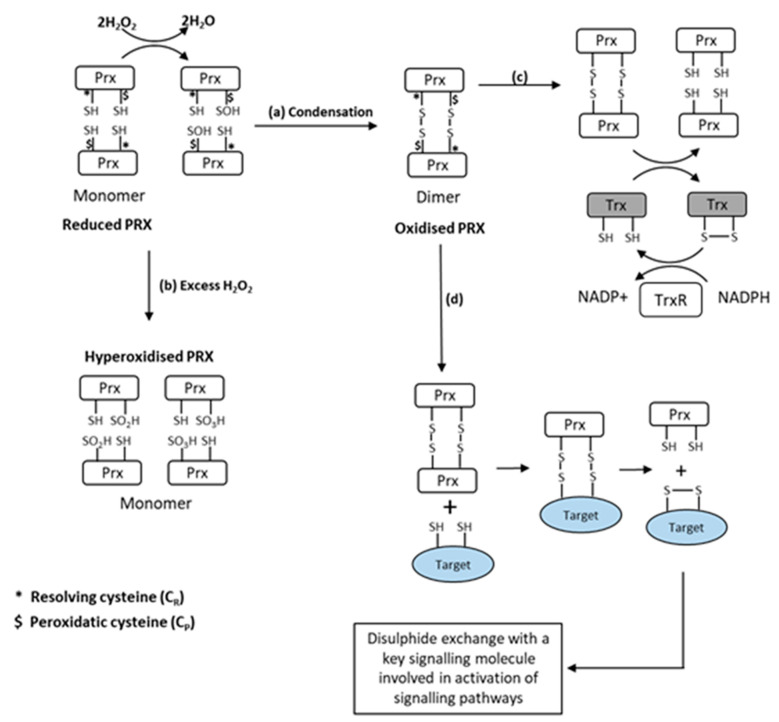
Schematic representation of the oxidation of 2-Cys Prx by H_2_O_2_. (**a**) Initial oxidation of Prx peroxidatic cysteine to form sulfenic acid with condensation to form Prx homodimers. (**b**) With further oxidation by H_2_O_2_, sulfinic or sulphonic acids are formed, which cannot form dimers (a process called hyperoxidation). (**c**) Dimerised Prx can be reduced by thioredoxin (Trx) at the expense of NADPH. (**d**) Dimerised Prx can also interact with reduced Cys in other proteins (indicated in grey), forming mixed dimers with transfer of oxidizing equivalents to the target protein from [64].

Prx reduce hydroperoxides to water and are classified by the number of cysteine (Cys) residues involved in the peroxidase activity. The 2-Cys Prx form a disulphide bond by reacting with peroxides [58]. Prx are several orders of magnitude more reactive with H_2_O_2_ than the typical redox-responsive signalling proteins [37]. Winterbourn calculated the selectivity of H_2_O_2_ for Prx in comparison with redox-sensitive signalling proteins such as PTPs and reduced glutathione (GSH) at approximate cellular concentrations, and concluded that Prx reacts with 99.9+% of the peroxide [59], further stating that “the oxidation (of PTPs and related enzymes) observed in cells is likely to be an indirect effect of peroxide reacting with a primary sensor”. Some studies have also recently indicated that Prx can function as a signal peroxidase to activate specific pathways [37,60]. A schematic illustrating the oxidation pathways for Prx is shown in Figure 2.

Prx also undergo other post-translational modifications in addition to the oxidation and formation of homodimers, including phosphorylation, acetylation, glutathionylation, and nitrosylation [61], which have been proposed to influence peroxidase activity and may play a role in signalling [61,62]. Finally, Prx become hyperoxidised by higher levels of H_2_O_2_, and this process has also been claimed to be important in regulating different cell signalling pathways [63] (Figure 2).

**Figure 3:**
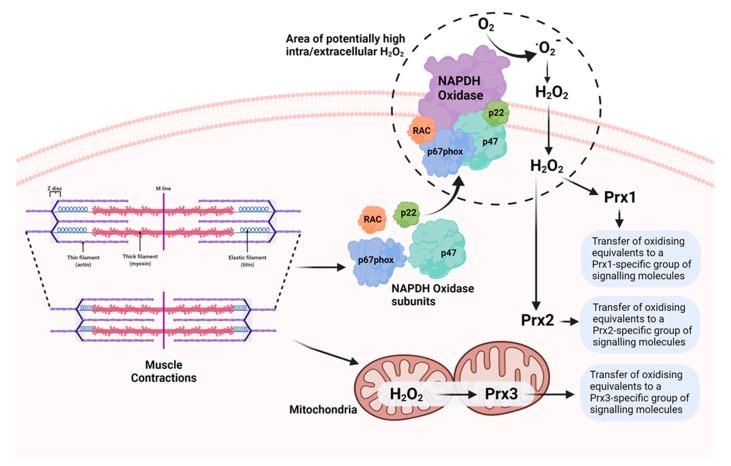
Potential mechanisms underlying generation of ROS (superoxide and H_2_O_2_) during muscle contractions and oxidation of 2-Cys Prx in different cellular compartments, leading to activation of specific groups of Prx-interacting signalling molecules involved in muscle adaptations to the contractile activity.

We examined the oxidation of Prx1, 2, and 3 by treatment with H_2_O_2_ and by contractile activity in isolated intact muscle fibres from mice. Low concentrations of H_2_O_2_ caused oxidation and dimerization of all three Prx, but higher concentrations induced the formation of hyperoxidised proteins that did not form dimers (Figure 2). The effects of contractile activity were also examined in isolated mouse FDB muscle fibres. Following commencement of contractile activity in fibres from adult mice, Prx1, 2, and 3 became oxidised and formed dimers when analysed on non-reducing Western blots [64]. Prx1 and 2 are localised to the cytosol of skeletal muscle, while Prx3 is found in the mitochondria. The contraction protocol used induces subsequent adaptations, including increased expression of cytoprotective and “antioxidant” proteins in skeletal muscle. Prx2 oxidation, in particular, occurred very rapidly, and was seen within 12 contractions (1 min in total), and oxidation of Prx1, 2, and 3 was seen by 15 min. Furthermore, Prx oxidation was rapidly reversed following the end of contractions. No hyperoxidation of Prx was seen. In contrast, our previous work found no evidence for a similar effect on Trx oxidation [65]. Thus, our data are compatible with the hypothesis that all three 2-Cys Prx isoforms are oxidised by physiological concentrations of H_2_O_2_ generated in skeletal muscle during contractile activity. Furthermore, the location of Prx3 in the mitochondria in comparison with Prx1 and 2 (cytosolic) supports the possibility that oxidation of different Prx may mediate specific adaptations to contractile activity depending on their location and interacting proteins (Figure 3). In very recent studies [66], we have examined the oxidation of human peroxiredoxins (Prdx) and redox-sensitive cysteines in peptides of proteins from muscle biopsies of volunteer human subjects prior to and following repeated periods of high-intensity interval training (HIIT) exercise. These studies indicated rapid oxidation of Prdx3 during muscle exercise, as previously seen in mice, but failed to show any contraction-induced oxidation of Prdx1 and 2. The reasons for these differences between the mouse and human data are currently unclear.

## 8. Effect of Ageing on Prx Oxidation in Contracting Skeletal Muscle Fibres

We also examined the effect of contractile activity on muscle Prx oxidation in muscle from old mice [64]. The baseline level of oxidation of Prx2 was significantly lower in FDB fibres from old compared with that from adult mice. Oxidation of the protein also occurred over a slower time course in fibres from old compared with adult mice, and the proportion of Prx2 in the oxidised form remained lower than the adult throughout. Prx1 and Prx3 oxidation in fibres from old mice were unchanged in comparison with that from adult mice [64].

## 9. Redox-Regulated Pathways Underpinning Adaptations to Contractile Activity in Skeletal Muscle Are Attenuated during Ageing

Failure of adaptations to stress occurs in many cellular models of ageing and tissues of old organisms [67]. In skeletal muscle, acute stress responses [68], mitochondrial biogenesis [19,69,70], and anabolic responses [71] in response to exercise are reduced by ageing. These age-related changes appear to reduce the efficacy of exercise in maintaining muscle, and previous data indicate that transgenic correction of specific responses to exercise help maintain muscle mass and function in old age [72,73,74]. A chronic increase in mitochondrial H_2_O_2_ generation has been claimed to cause the attenuation of redox-mediated adaptations to contractile activity in ageing. A chronic increase in ROS activities was found in muscle from old mice at rest, with no further increase following contractions [75], and we speculated that this was caused by increased mitochondrial ROS generation [46]. Mice with knockout of Cu, Zn superoxide dismutase (SOD1null mice), a model of accelerated muscle ageing in which muscle mitochondrial ROS generation is also elevated [76], also show attenuated responses to contractions. Martinez-Guimera et al. developed a molecular model in which they described a process termed “molecular habituation”, which may help to explain the attenuation of responses. They characterised this as “a sustained ROS signal which reduced the responsiveness of signalling pathways through prolonged activation of negative regulators”, such as has been reported to occur in ageing with upregulation of regulatory proteins for ROS, including catalase, glutathione peroxidase 1, thioredoxin (Trx)1 and 2, and peroxiredoxins (Prx) 3–6 [77]. In a recent hypothesis article, we have reviewed this area and argued for an alternate mechanism for the attenuation of redox-regulated responses to contractile activity, in which key protein cysteine thiols become increasingly reduced (i.e., less oxidised) with ageing [78].

## 10. Denervation of Individual Muscle Fibres Leads to Increased Mitochondrial Peroxide Generation during Ageing

Loss of both motor units and innervation of individual fibres has been reported in muscles from older humans and animals [20,21]. Transection of the innervating nerve caused a large increase in muscle mitochondrial peroxide generation [79], and these studies identified a key role for motor neuron and NMJ integrity in the regulation of muscle mitochondrial ROS generation in old mice. Furthermore, partial denervation of the mouse TA muscle caused an increase in mitochondrial peroxide generation in the denervated fibres and also in adjacent innervated fibres [80]. These data suggest that loss of innervation in fibres contributes to increased mitochondrial ROS generation [80] and associated mitochondrial degeneration [81] in ageing.

Skeletal muscle mitochondria exist in a complex reticulum [82] that is hypothesised to rapidly distribute energy through direct coupling of mitochondrial membrane potential to drive local ATP production. The reticulum acts as a ‘power-grid’, forming subnetworks that are proposed to effectively act as circuit breakers within the interconnected organelle, preventing the propagation of local dysfunction while preserving energetic homeostasis [82,83]. The structure and distribution of mitochondrial networks are extremely dynamic and are regulated by several key processes, including mitochondrial fission, fusion, biogenesis, and mitophagy [84]. Under physiological conditions, mitochondrial fission and fusion events occur in a balanced frequency to maintain not only the size and shape of the mitochondrial network, but also its gross distribution. Fusion results in the elongation of mitochondria into interconnected, tubular networks, enabling the mixing of their contents (i.e., metabolites, proteins, and mtDNA) and the redistribution of energy [82,85]. Furthermore, a fused network is thought to prevent the local accumulation of dysfunctional mitochondria [82,86]. In contrast, mitochondrial fission is a process that acts to fragment the network into smaller, discrete organelles [87]. Fission appears to segregate network components, which may be damaged or dysfunctional, for removal by mitophagy. Together with mitochondrial biogenesis, these processes ensure restructuring of the reticulum in response to stimuli, including nutrient availability, cellular stress, and other molecular signals. The mitochondrial reticulum is closely associated with and attached to the muscle cytoskeleton, and the reticulum structure is determined by factors including the regulation of dynamin-like proteins on individual mitochondria [88], interactions between mitochondria and organelles, such as nuclei and sarcoplasmic reticulum [89], and direct connections between mitochondria and the cytoskeleton [90].

Disuse, such as with exposure of muscle to microgravity or extended bed rest, induces disorganisation of the muscle cytoskeleton and triggers mitophagy, implicating a mechanistic link between cytoskeletal dynamics and mitochondrial content [91], and space-flown rats showed an aberrant distribution of mitochondria and a decrease in the expression of cytoskeletal genes in muscle [92]. Immobility, or extended bed rest, also lead to increased muscle mitochondrial ROS production [93]. Increased mitochondrial ROS, low respiration rates, and increased apoptosis are known to be consequences of the reorganisation of the mitochondrial reticulum via increased fission and reduced fusion [94,95,96].

**Figure 4:**
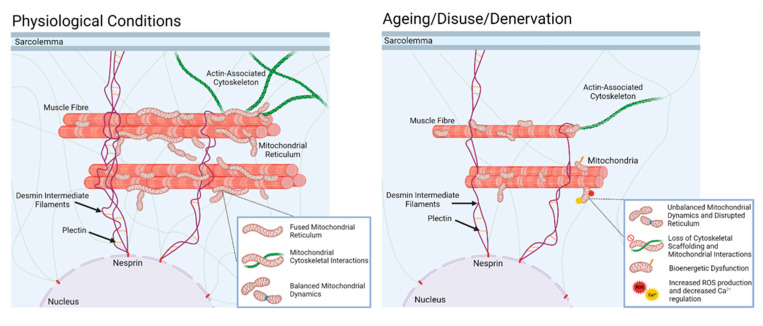
In normal physiology, skeletal muscle mitochondria are maintained in a complex reticulum by interactions with the actin cytoskeleton and intermediate filaments. This condition is associated with balanced mitochondrial dynamics, high capacity for energy production, low mitochondrial peroxide production, and minimal apoptotic signalling. With disuse of the muscle, or, as we speculate, denervation associated with ageing, the supportive interactions with the cytoskeleton are lost, leading to disruption of the reticulum, a change in mitochondrial dynamics (increased fission, reduced fusion, and increased mitophagy), and increased degenerative signalling through reduced energy production, increased mitochondrial peroxide, and increased apoptotic signalling.

Studies performed in models of ageing suggest that mitochondria show a change to a phenotype reflecting modified fusion, together with a change in orientation more perpendicular to the fibre axis [97] in association with other changes in mitochondrial dynamics [98]. We speculate that during ageing, the repeated episodes of muscle denervation led to a loss of cytoskeletal organisation and caused disruption of links to mitochondria that may be a pre-requisite for mitochondrial disruption and increased mitochondrial peroxide generation (Figure 4).

## 11. Conclusions

Research into the generation of ROS by contracting skeletal muscle came to prominence in the 1980s [30,99], and in the intervening ~30 years, many key aspects of the processes and their consequences for muscle health and vitality have been identified. This has had multiple consequences in terms of our understanding of the need for (antioxidant) supplements and optimum training regimens to maintain redox homeostasis. Recent studies are revealing fundamental, but subtle, interacting roles of ROS in modulating multiple aspects of muscle responses to physiological and pathological stimuli. Obtaining a full understanding of these processes offers great potential for manipulation of redox-regulated processes to modify and maintain skeletal muscle mass and function in ageing and multiple disease states.

### 11.1. Are There Species Differences in Redox Regulation of Muscle Responses to Exercise?

The vast majority of basic studies of ROS and redox-mediated processes in skeletal muscle have been undertaken in rodent (usually mouse) models, or even in cell culture. Considering the differences in the whole body and skeletal muscle physiology between quadruped rodents and biped humans, it is appropriate to consider how relevant studies of exercising rodents or contracting rodent muscles are to understanding redox regulation of muscle responses in humans. Key studies over many years have shown that fundamental processes of muscle contraction are essentially comparable across mammalian species, but can this be extrapolated to studies of, for example, the effects of antioxidant supplements on muscle function from the rodent to humans? Three examples from the work described above will be discussed to illustrate the issues involved and provide support for the relevance of rodent data to understand human muscle redox processes.

### 11.2. Role of NADPH Oxidase in Mediating Redox Responses to Exercise

As previously discussed, initial studies indicated that mitochondrial generation of ROS might predominate during muscle contractile activity [30], but subsequent detailed studies in isolated mouse fibres indicated activation of NADPH oxidase to be the predominant source over short periods of contractile activity [31]. There is considerable interest in the potential role of muscle-derived ROS in regulating skeletal muscle glucose metabolism [100,101], and Jensen and Henriquez-Olguin [102,103,104] have utilised state-of-the-art approaches to demonstrate a role for muscle NADPH oxidase in mediating adaptations in muscle glucose metabolism. They showed a role for NADPH oxidase in studies of exercise in mice and examined ROS generation in human muscle samples using a DCFH oxidation approach. These data appear to confirm the increase in ROS activities in human vastus lateralis muscle that follows treadmill running.

### 11.3. Redox-Related Adaptations to Exercise in Muscle of Elderly Subjects

Cobley and colleagues worked with our group to examine the influence of age and training status on the redox-related adaptations of human muscle to exercise [19]. They took biopsies from the vastus lateralis muscle of healthy volunteers and examined the expression of HSPs, antioxidant enzymes, and NO synthase isoenzymes before and after HIIT exercise. They included groups of young and older volunteers with differing lifelong exercise training status. We concluded that in the untrained state, exercise in the older subjects did not upregulate multiple proteins, in comparison with that seen in younger subjects, but lifelong training preserved some but not all (e.g., SOD2, HSP72, PRX5) of the exercise responses. From this study, we argued that the data support many, but not all, findings from previous animal studies, and suggested that there are parallel ageing effects in humans and mice at rest and after exercise that are not corrected by lifelong training in human skeletal muscle.

### 11.4. Oxidation of Muscle Protein Cysteine Thiols Following Exercise in Human Subjects

In a complementary study to that described above, Pugh and colleagues used a muscle biopsy approach and redox proteomics to study vastus lateralis muscles of adult (18–30 years) and old (64–79 years) male and female subjects who undertook high-intensity cycling exercise. This consisted of 5 sets of 2 min intervals performed at 80% maximal aerobic power output (PPO), with 2 min recovery cycling at 40% PPO between sets [66]. Samples of muscle were taken prior to and immediately following the first, second, and fifth high-intensity intervals. Analysis of redox cysteines indicated five cytosolic proteins in older subjects with lower oxidation (i.e., greater reduction) than that in young adults. Muscle peroxiredoxin 3 (Prdx3) oxidation occurred rapidly in response to exercise in both adult and older subjects, supporting the possibility that Prdx3 is a key effector protein for mitochondrial redox signalling. These data are similar to those previously reported in mice (e.g., see [78]), but this human study also revealed that overall, redox homeostasis was well-maintained in adult subjects following exercise, but with a significant increase in oxidation of multiple mitochondrial and cytosolic protein cysteines in old subjects.

It is apparent for these three brief examples that many fundamental aspects of the muscle redox signalling are conserved between rodents and humans, but detailed aspects of the processes may not always be identical. There is some evidence that this may be related to the training state of the subjects, with some ageing changes being reversed (or prevented) by regular training exercise [19]. Training status is very rarely modified in essentially sedentary laboratory rodents, and there is concern in attempting to make cross-species comparisons because exercise protocols used in the two species may not be comparable. Further studies are required to address these issues.

## Data Availability

Not applicable.
